# Methods for analyzing tellurium imaging mass cytometry data

**DOI:** 10.1371/journal.pone.0221714

**Published:** 2019-09-03

**Authors:** Jay Bassan, Mark Nitz

**Affiliations:** 1 Department of Chemistry, University of Toronto, Toronto, Ontario, Canada; 2 BIMDAQ Ltd, Bexleyheath, United Kingdom; Fondazione Pisana per la Scienza, ITALY

## Abstract

Imaging mass cytometry (IMC) is a technique allowing visualization and quantification of over 40 biological parameters in a single experiment with subcellular spatial resolution, however most IMC experiments are limited to endpoint analysis with antibodies and DNA stains. Small molecules containing tellurium are promising probes for IMC due to their cell permeability, synthetic versatility, and most importantly their application to sequential labelling with isotopologous probes (SLIP) experiments. SLIP experiments with tellurium-containing probes allow quantification of intracellular biology at multiple timepoints with IMC. Despite the promise of tellurium in IMC, there are unique challenges in image processing associated with tellurium IMC data. Here, we address some of these issues by demonstrating the removal of xenon background signal, combining channels to improve signal-to-noise ratio, and calculating isotope transmission efficiency biases. These developments add accuracy to the unique temporal resolution afforded by tellurium IMC probes.

## Introduction

The emerging technology of imaging mass cytometry (IMC) has delivered insight into many aspects of biology including the heterogeneity of breast cancer tumours and the tissue distribution of cisplatin. [1, 2] Tissue sections are stained with more than 40 different antibodies, each of which is conjugated to a polymer that chelates a distinct elemental isotope, typically from the lanthanide series. The section is then ablated pixelwise by a rastering, pulsed ultraviolet laser. The ablated material from each pixel passes into an inductively-coupled plasma mass spectrometer and each isotope, which corresponds to a specific antibody, is quantified. A multi-channel image is thereby created with many more channels accessible than traditional immunohistochemical or immunofluorescent optical imaging. This technique has been limited to the imaging of static biological markers (i.e. proteins), DNA, or certain small molecule probes against which custom antibodies have been raised (e.g. EF5). [1, 3] With tellurium as an IMC-visible element, we have developed tellurophenes as biologically-compatible mass tags whose imaging requires no antibody staining. [4] Probes for specific biological processes can be synthesized by linking tellurophenes, which are aromatic, stable, and non-toxic, to activity-based functional groups that covalently bind cells of a certain phenotype. In this way, IMC can be used to visualize processes or microenvironments including protein synthesis and cellular hypoxia. [5, 6] Imaging of these compounds is not dependent on antibody-epitope binding and as such represents direct visualization of the probe itself. Tellurium exists naturally as a mixture of eight stable isotopes, of which six are commercially available in an isotopically-enriched form. If a robust probe is developed and synthesized in isotopically-enriched variants, its target biochemical process may be investigated with spatial and temporal resolution with IMC. For example, changes in hypoxia over time were quantified by dosing one isotope of a tellurophene conjugated to a 2-nitroimidazole group, followed by waiting or an intervention, followed by a dose of the same molecule containing the second isotope. [6] The single mass unit resolution of IMC then allows quantification of the difference in labelling of the probes. We term this approach sequential labelling of isotopologous probes (SLIP). SLIP experiments with tellurium add temporal resolution to the IMC toolbox, which is crucial in understanding the deep biological profile imaged by IMC.

Despite the specific benefits of tellurium probes, the element brings unique data processing challenges. Contamination with overlapping isotopes of xenon can cause significant background signal, and differences in detection efficiency across isotopes may reduce the accuracy of SLIP experiments. In addition, when natural abundance tellurium probes are used, it is possible to improve the signal-to-noise ratio by combing signals across multiple mass channels. While software for analyzing IMC data exist, [7, 8] they do not deal explicitly with tellurium as an analyte and so do not treat these challenges. Given the specific benefits of tellurophene probes, we sought to design data processing strategies that would improve the accuracy of research in this field and allow SLIP experiments to be included as robust methods in the IMC community.

## Materials and methods

### Natural abundance intestine image

We used IMC data from our previous publication detailing a tellurium-containing probe for protein synthesis, where the experiment is described in detail. [5] Briefly, mice were injected with TePhe (60 mg kg^−1^, intravenous) and sacrificed three hours later.

### SLIP experiment tumour image

We used IMC data from our previous publication using SLIP experiments to probe dynamic hypoxia, where the experiment is described in detail. [6] Briefly, mice were injected with isotopically-enriched ^125^Te (60 mg kg^−1^, intravenous) then after 24 hours injected with ^122^Te (60 mg kg^−1^, intravenous).

### Tissue and slide preparation

The tissue of interest was excised, formalin fixed, paraffin embedded, and cut into 5 μm sections. The slides were processed for IMC following the standard protocol (www.fluidigm.com, protocol ID PN 400322 A3). Stained tissue sections were imaged on the Hyperion™ Imaging System at 200 Hz.

## Results and discussion

### Removing xenon background

**Fig 1:**
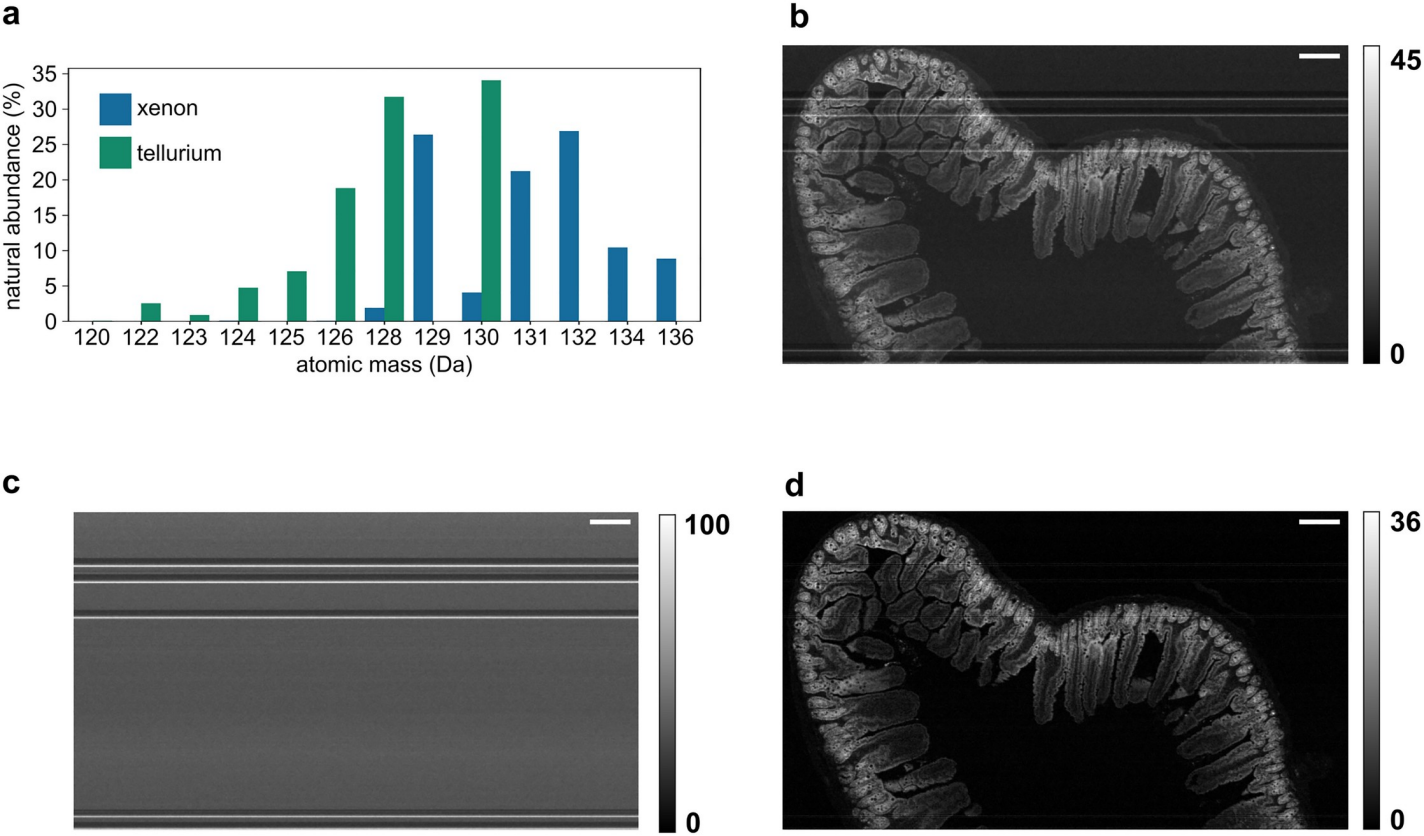
Xenon background is removed from tellurium images of mouse jejunum without blank sample runs. (a) Xenon has several isotopes whose masses overlap with tellurium. 128 and 130 are the most obfuscated mass channels, but ^124^Xe (0.095% abundant) and ^126^Xe (0.089% abundant) also contaminate tellurium significantly. (b) The 128 mass channel image, comprising ^128^Te and ^128^Xe. (c) The 134 mass channel image, comprising only ^134^Xe. This image is used to estimate the ^128^Xe image. (d) The corrected 128 mass channel image, now representing only ^128^Te. Colour bars represent IMC counts in arbitrary units. Scale bars 200 μm.

Xenon has several naturally-occuring isotopes that overlap in mass with the heavier tellurium isotopes (Fig 1a). The argon used in IMC can be contaminated with xenon and as a result some mass channels used to monitor tellurium contain signal from xenon. The amount of xenon in the argon flow varies over time, potentially due to a non-heterogeneous mixture of the gases in the storage vessel but further experiments are required to determine the source of this unexpected contamination. In a phenomenon that is especially disruptive to imaging, large ‘spikes’ of xenon are drawn in to the mass cytometer and result in streaks of background signal across a tellurium image (Fig 1b).

To solve this issue, we recorded the 134 atomic mass unit (AMU) image, **X**, which contains xenon but no tellurium, to quantify the xenon in each pixel (Fig 1c). Next we used the known isotopic ratios of ^134^Xe to the xenon contaminating isotope of interest (in this case ^128^Xe) to estimate an image corresponding to the xenon component of the 128 AMU channel, **Y**. By considering that the observed image from the 128 AMU channel, **I**, is a sum of the xenon component **Y** and the tellurium component **T** (i.e. the true ^128^Te image), **T** can be calculated by subtracting **Y** from **I**:

$$\begin{array}{c} I=T+Y \end{array}$$ (1)

$$\begin{array}{c} Y=X\times \frac{\text{abundance}{(}^{128}\text{Xe})}{\text{abundance}{(}^{134}\text{Xe})} \end{array}$$ (2)

$$\begin{array}{c} T=I-X\times \frac{\text{abundance}{(}^{128}\text{Xe})}{\text{abundance}{(}^{134}\text{Xe})} \end{array}$$ (3)

Xenon background subtraction returns an image that represents the corrected tellurium image (Fig 1d). In addition to allowing more accurate visual inspection of images, xenon background subtraction is critical in SLIP experiments where the accurate quantification of each tellurium isotope is important to downstream data analysis. Xenon background may also be removed more precisely by measurement of xenon spillover into tellurium channels to calculate a spillover coefficient instead of relying on theoretical natural abundance values. [9] However, our method requires no measurement of blank samples and therefore uses less instrument time, which is an important consideration in IMC (≈ 90 minutes to acquire a 1 mm × 1 mm image). To ensure that our approach of using theoretical isotope ratios is valid, we calculated the actual ratio of ^128^Xe to ^134^Xe in a region of the image that contained no tissue (and therefore no tellurium). We found that the theoretical ratio (0.183) is comparable to the calculated ratio (0.180), confirming that xenon removal can be effected without measurement of blank samples.

### Calculating bias in isotope transmission efficiency

Although IMC is a quantitative technique, systematic error exists in the sensitivity of the detector to ions of different mass. [10] As a result, the instrument is more sensitive to, for example, ^126^Te ions than ^124^Te ions. While this is of little consequence in natural abundance experiments, SLIP experiments rely on quantifying differences between mass channels and therefore require correction of this transmission efficiency bias (TEB).

If the instrument is equally sensitive to all isotopes, the relative intensity of the pixel at location *ij* from the ^a^Te image **A** to the pixel at the same location from the ^b^Te image **B** would depend only on the relative isotopic abundances:

$$\begin{array}{c} \frac{A_{ij}}{B_{ij}}=\frac{\text{abundance}{(}^{\text{a}}\text{Te})}{\text{abundance}{(}^{\text{b}}\text{Te})} \end{array}$$ (4)

The amount of deviation from (4) represents the TEB. We took advantage of data from natural abundance tellurium to calculate the TEB between two isotopes of interest, ^a^Te and ^b^Te. A ratio image **R** is calculated, in which pixel **R**_*ij*_ is the ratio of the corresponding pixels in the two isotope images of interest, **A**_*ij*_ and **B**_*ij*_:

$$\begin{array}{c} R_{ij}=\frac{A_{ij}}{B_{ij}} \end{array}$$ (5)

The median value of the pixels in the ratio image **R** is compared to the theoretical ratio of isotope a to isotope b to give the TEB:

$$\begin{array}{c} \text{TEB}=\text{median}\left(R\right)\times \frac{\text{abundance}{(}^{\text{a}}\text{Te})}{\text{abundance}{(}^{\text{b}}\text{Te})} \end{array}$$ (6)

We chose to use a ratio-based approach as opposed to linear regression to avoid the designation of one isotope as the dependent variable and the other as the independent variable. Pixels with a value of 0 in either image must be excluded to avoid division by 0. Non-zero but low pixel values, while mathematically sound in TEB calculation, skew results by creating unreasonably high or low values in **R**. We found a threshold of 1 count per pixel to allow robust quantification of the TEB. In addition, a moderate Gaussian blur (*σ* = 1 μm) aids in removing outlier pixels from the analysis. As anticipated, removal of xenon background is necessary before calculation of TEB values.

**Fig 2:**
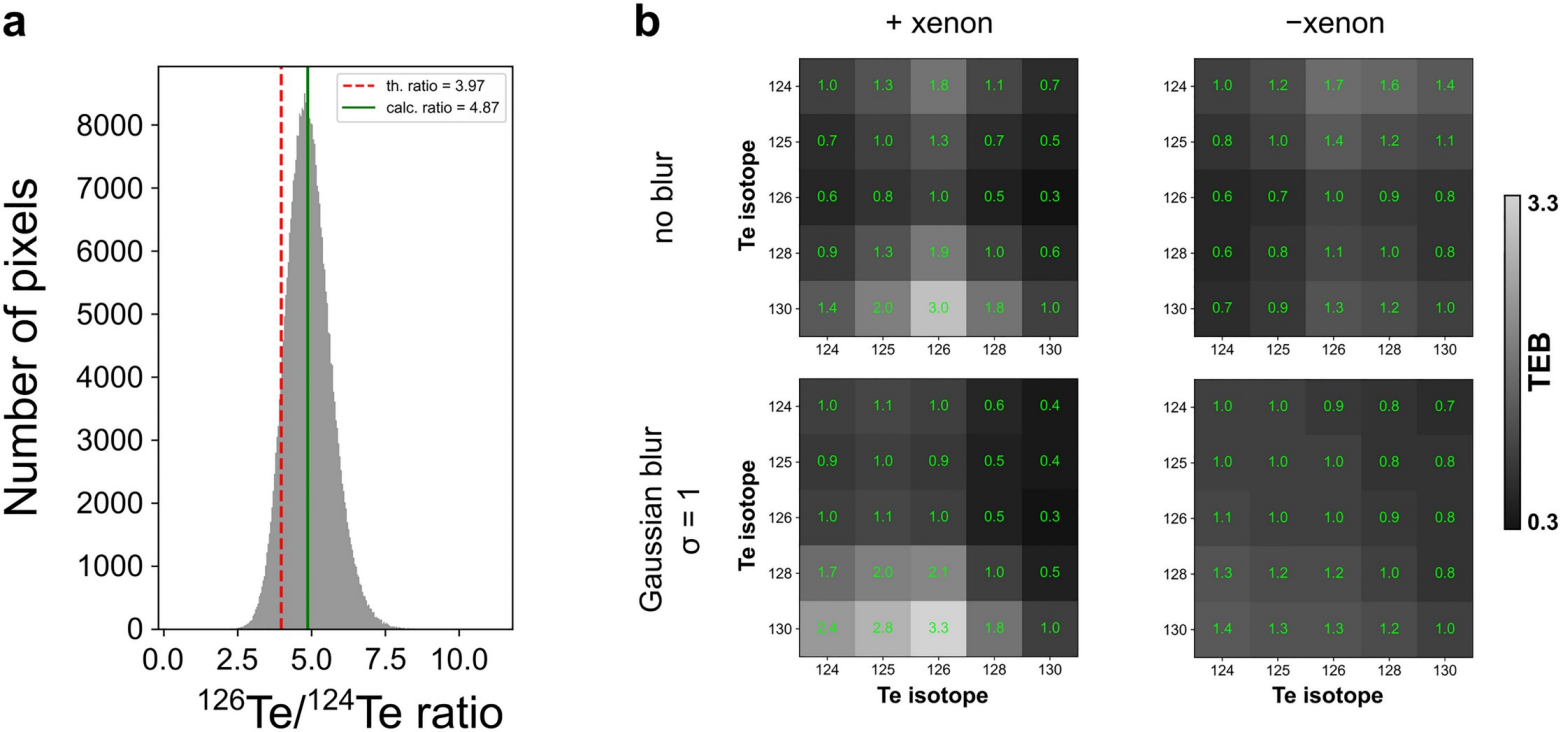
Transmission efficiency bias is calculated from a natural-abundance tellurium image. (a) The ^124^Te and ^126^Te channels of the same image are compared to quantify the transmission efficiency bias (TEB). The histogram represents pixel values obtained by dividing the ^126^Te image by the ^124^Te image. The theoretical ratio (based on isotopic abundances) is shown along with the calculated ratio. (b) Heatmaps displaying the TEB for different pairs of isotopes. TEB values were calculated with or without xenon removal, and with or without Gaussian blurring of the images.

We observed that heavier tellurium isotopes are overdetected by the mass cytometer (Fig 2). This effect has two contributing causes. First, ions with lower mass have lower kinetic energy in the ion beam that travels from the plasma torch to the time-of-flight measurement chamber and have a higher chance of being ejected from the beam before detection. [11] Second, the mass cytometer is equipped with a quadrupole mass filter that removes ions with *m*/*z* > 80 Da and is tuned to transmit ions with *m*/*z* ≈ 160 Da most efficiently. [10] Therefore the lower mass Te ions, being more easily ejected and further from the target of the mass filter, are transmitted less efficiently.

Given that the TEB is a property of the instrument, it is important that users calculate their own values for the TEB between two isotopes, preferably shortly before and after the experiment whose data is to be corrected, since the mass cytometer is known to drift over time, [12] and individual mass cytometers may have different patterns of sensitivity. [10]

**Fig 3:**
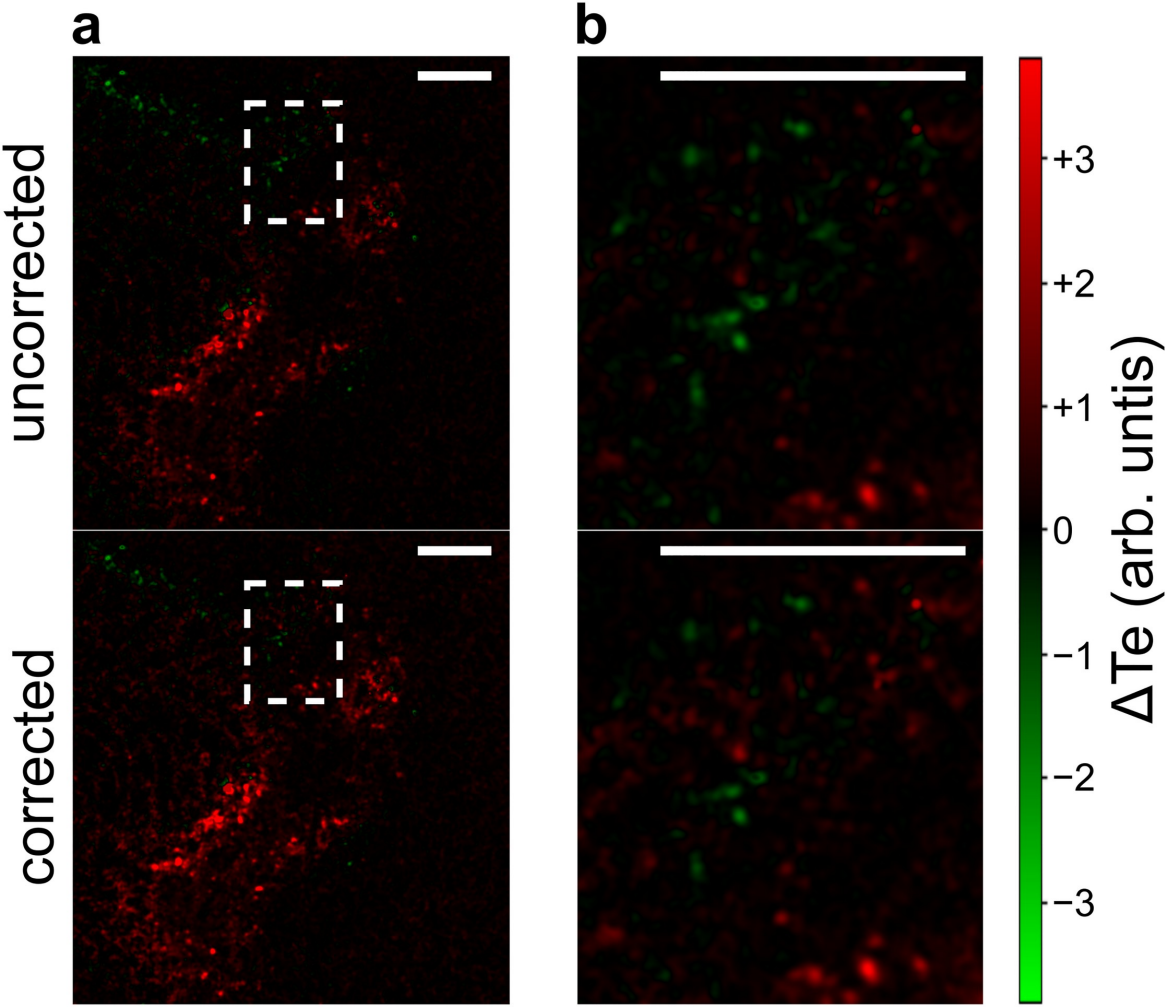
TEB correction is important for quantification of SLIP experiments. Data from a SLIP experiment measuring changes in hypoxia over time. (a) The same region of a tumour showing increasing hypoxia in red and decreasing hypoxia in green. The top panel shows the data before TEB correction and the bottom panel shows the data after TEB correction. (b) Expansion of the boxed region in (a) highlighting some regions that change colour with TEB correction. Scale bars 200 μm.

We applied TEB correction to data from a published SLIP experiment investigating changes in tumour hypoxia over time (Fig 3). [6] In the experiment, mice bearing PANC-1 xenografts were dosed with two isotopologues of a compound that labels hypoxia 24 hours apart. Tumour sections were analyzed by IMC and a difference image was created, highlighting increasing hypoxia in red and attenuating hypoxia in green. After TEB correction, the conclusion of the experiment—that cycling hypoxia in tumours occurs without intervention—does not change. However, 8.5% of pixels change colour with TEB correction, indicating a change in the interpretation of these cells’ hypoxic shifts. Therefore, TEB correction is an important step in SLIP experiment analysis.

### Combining tellurium channels for improved signal-to-noise ratio

While SLIP experiments add temporal resolution to IMC, single-timepoint experiments and validation of new probes are carried out with natural abundance tellurium, which has six isotopes with abundances above 1%. Experiments with natural abundance tellurium may therefore combine images of multiple tellurium isotopes together to improve signal-to-noise ratio (SNR) and reduce artefacts in the image. There are several possible approaches to combining the tellurium channels. The simplest and most intuitive approach is to sum the channels arithmetically. While this approach is minimally manipulative, it does not effectively remove background noise. This is because noise in IMC is not distributed around 0, and can only have positive values. Therefore when adding channels together, the noise accrues instead of cancelling. An alternative method is taking the product of n tellurium channels, then normalizing by taking the nth root of the product (i.e. the geometric mean). The geometric approach is more effective at removing background noise from the image because a pixel value of 0 in any channel will result in a pixel value of 0 in the output image. However, if a very low signal image is included in the geometric mean, the output will be unnecessarily skewed and contain many pixels with a value of 0. Typically, ^130^Te, ^128^Te, and ^126^Te can all be included without skewing the image to 0, since they are the highest abundance isotopes, but users should decide empirically which isotopes to include.

**Fig 4:**
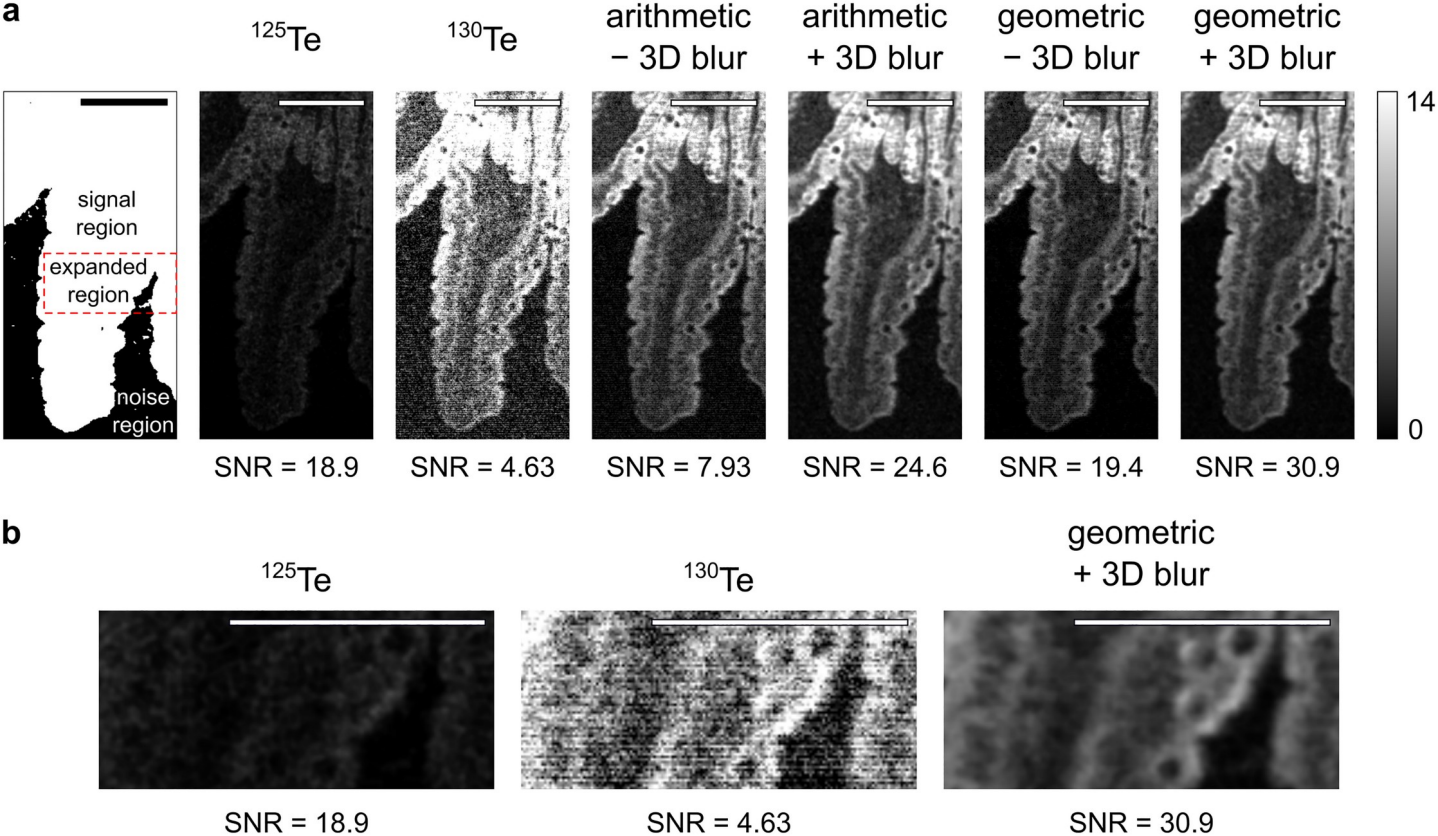
Signal-to-noise ratio is improved by combining tellurium channels. (a) Combination of ^125^Te, ^126^Te, ^128^Te, and ^130^Te images. The signal and noise regions are defined arbitrarily based on knowledge of the tissue of interest. Single isotope images may have either low signal (e.g. ^125^Te) or high noise (e.g. ^130^Te). The effect of combining the images in an arithmetic or geometric fashion, with or without 3-dimensional Gaussian blurring (*σ* = 1) is shown. (b) Images from ^125^Te, ^130^Te, or the geometric combination with blur of the expanded region marked in (a). SNR denotes signal-to-noise ratio. Scale bars 100 μm.

A further option is to stack each image to be combined into a 3-dimensional array and perform a 3-dimensional Gaussian blur. Then the blurred 3-dimensional array can be collapsed into a 2-dimensional image in either an arithmetic or geometric fashion. We compared the SNRs of an image processed arithmetically against one processed geometrically, both with and without 3-dimensional Gaussian blur (Fig 4). We define SNR as:

$$\begin{array}{c} \text{SNR}=\frac{\mu_{s}}{\sigma_{n}} \end{array}$$ (7)

Where *μ*_*s*_ is the mean intensity in an arbitrarily-defined signal region of the image, and *σ*_*n*_ is the standard deviation of the intensity in an arbitrarily-defined noise region of the image. In this case, the signal region was defined as the region containing tissue mounted on the microscope slide and the noise region as the region containing only the microscope slide.

We found that a three-dimensional Gaussian blur provides a strong improvement on the SNR in both arithmetic and geometric modes, but geometric combination improves the SNR more substantially than arithmetic combination. Blurring may improve SNR but will also reduce spatial resolution; we expect that each experiment will have an optimal balance between SNR and resolution that depends on the specific biology being interrogated. Improvement of the SNR of IMC experiments with this technique will be useful for any experiment involving natural abundance tellurium probes. This technique may even find application in improving the SNR in imaging when more than one type of probe against the same target has been employed.

## Conclusion

We have described strategies that enhance the analysis of IMC experiments that use tellurium probes. Xenon background signal, which affects the quality of tellurium IMC data, was effectively removed by subtraction of a pure xenon channel modified according to known isotopic distributions. The detection bias of different isotopes of tellurium—which could adversely affect a SLIP experiment—was quantified and corrected for, based on measurement of natural abundance samples. Finally, we leveraged the polyisotopic nature of tellurium to improve SNR by combining images from different mass channels.

We expect that the techniques described here will become part of the routine IMC workflow when tellurium probes have been used. Coupled with the expanding toolkit of tellurium probes, we hope to progress the study of dynamic biology in vivo.

We have implemented the strategies described here, as well as other general IMC processing methods, in a Python package available at https://github.com/jaybassan/teimc and PyPI (teimc). The package is concise, readable, and amenable to customization; it is open source and based on other open-source libraries. The IMC data presented in this manuscript are from our previous studies and are deposited on the Open Science Framework (https://osf.io/rxez7/); [5, 6] xenon removal and channel combination were applied to data published in reference [5].

We continue the development of software as a complement to laboratory advances, and especially hope to develop cell segmentation solutions coupled with automated SLIP analysis.
